# Epidemiology of Human Seasonal Coronaviruses Among People With Mild and Severe Acute Respiratory Illness in Blantyre, Malawi, 2011–2017

**DOI:** 10.1093/infdis/jiad587

**Published:** 2024-08-16

**Authors:** Dory Kovacs, Ivan Mambule, Jonathan M. Read, Anmol Kiran, Moses Chilombe, Thandiwe Bvumbwe, Stephen Aston, Mavis Menyere, Mazuba Masina, Moses Kamzati, Thokozani Namale Ganiza, Danielle Iuliano, Meredith McMorrow, Naor Bar-Zeev, Dean Everett, Neil French, Antonia Ho

**Affiliations:** 1 School of Biodiversity, One Health and Veterinary Medicine, University of Glasgow, Glasgow, United Kingdom; 2 Malawi-Liverpool-Wellcome Trust Clinical Research Programme, University of Malawi College of Medicine, Blantyre, Malawi; 3 Research Department, Joint Clinical Research Centre, Kampala, Uganda; 4 Centre for Health Information Computation and Statistics, Lancaster Medical School, Lancaster University, Lancaster, United Kingdom; 5 Malaria Alert Centre, Kamuzu University of Health Sciences, Blantyre, Malawi; 6 Blantyre Malaria Project, Blantyre, Malawi; 7 Institute of Systems, Molecular and Integrative Biology, University of Liverpool, Liverpool, United Kingdom; 8 National Center for Immunization and Respiratory Diseases, Centers for Disease Control and Prevention, Atlanta, Georgia; 9 International Vaccine Access Center, Department of international Health, Bloomberg School of Public Health, Johns Hopkins University, Baltimore, Maryland, United States; 10 Department of Pathology and Infectious Diseases, College of Medicine and Health Sciences, Abu Dhabi, United Arab Emirates; 11 Infection Research Unit, Khalifa University, Abu Dhabi, United Arab Emirates; 12 Institute of Infection, Veterinary and Ecological Sciences, University of Liverpool, Liverpool, United Kingdom; 13 Medical Research Council-University of Glasgow Centre for Virus Research, University of Glasgow, Glasgow, United Kingdom

**Keywords:** human, seasonal coronavirus, severe acute respiratory illness, pathogen-attributable fraction, Malawi

## Abstract

**Background.:**

The aim of this study was to characterize the epidemiology of human seasonal coronaviruses (HCoVs) in southern Malawi.

**Methods.:**

We tested for HCoVs 229E, OC43, NL63, and HKU1 using real-time polymerase chain reaction (PCR) on upper respiratory specimens from asymptomatic controls and individuals of all ages recruited through severe acute respiratory illness (SARI) surveillance at Queen Elizabeth Central Hospital, Blantyre, and a prospective influenza-like illness (ILI) observational study between 2011 and 2017. We modeled the probability of having a positive PCR for each HCoV using negative binomial models, and calculated pathogen-attributable fractions (PAFs).

**Results.:**

Overall, 8.8% (539/6107) of specimens were positive for ≥1 HCoV. OC43 was the most frequently detected HCoV (3.1% [191/6107]). NL63 was more frequently detected in ILI patients (adjusted incidence rate ratio [aIRR], 9.60 [95% confidence interval {CI}, 3.25–28.30]), while 229E (aIRR, 8.99 [95% CI, 1.81–44.70]) was more frequent in SARI patients than asymptomatic controls. In adults, 229E and OC43 were associated with SARI (PAF, 86.5% and 89.4%, respectively), while NL63 was associated with ILI (PAF, 85.1%). The prevalence of HCoVs was similar between children with SARI and controls. All HCoVs had bimodal peaks but distinct seasonality.

**Conclusions.:**

OC43 was the most prevalent HCoV in acute respiratory illness of all ages. Individual HCoVs had distinct seasonality that differed from temperate settings.

Before the severe acute respiratory syndrome coronavirus 2 (SARS-CoV-2) emerged, 4 human coronaviruses (HCoVs) were known to circulate endemically, including 2 alphacoronaviruses, 229E and NL63, and 2 betacoronaviruses, OC43 and HKU1. HCoVs predominantly cause mild, but occasionally severe, respiratory tract infections [1]. They typically peak in colder months in temperate settings, and often co-circulate with one another [2, 3]. Two zoonotic betacoronaviruses have emerged in the past 2 decades: severe acute respiratory virus–associated coronavirus in 2002 [4] and Middle East respiratory syndrome coronavirus in 2012 [5]. Both caused severe but limited outbreaks. More recently SARS-CoV-2 [6, 7], also a betacoronavirus, emerged as a global pandemic. Coronavirus disease 2019 (COVID-19)–associated morbidity and mortality have been lower in Africa compared to other continents [8]. Several hypotheses have been postulated to explain this phenomenon, including younger populations, varying comorbidity profile, and differing surveillance strategies, as well as cross-protection from circulating coronaviruses [9, 10]. Tso et al [11] identified a higher prevalence of cross-reactive anti-SARS-CoV-2 antibodies in prepandemic specimens from Tanzania (19%) and Zambia (14.1%), compared to specimens from the United States (2.4%). Indeed, recent exposure to seasonal coronaviruses may be associated with milder COVID-19 [12, 13].

A detailed understanding of the epidemiology and seasonality of HCoV infections may provide insights into the future seasonality of SARS-CoV-2. Although a growing proportion of the global population has received COVID-19 vaccination, the future trajectory that SARS-CoV-2 will follow remains unclear. It is speculated that, similar to the spread of influenza A(H1N1) in 2009, SARS-CoV-2 will evolve to become an endemic virus and circulate seasonally [14, 15].

Information on the epidemiology of seasonal HCoVs and their contribution to acute respiratory infection, especially from Africa, has been limited to date [16-23]. We characterized the epidemiology and seasonal patterns of HCoVs 229E, OC43, NL63, and HKU1 in children and adults recruited from sentinel respiratory virus surveillance and a referral facility–based prospective observational study over 7 years in an urban setting in southern Malawi.

## METHODS

### Study Setting

The study setting has been described previously [24]. Respiratory viral surveillance was established in Blantyre, Malawi, following the 2009 influenza A(H1N1) pandemic in recognition of the fact that the epidemiology of respiratory viruses in Africa was poorly understood. Between January 2011 and December 2017, we conducted sentinel surveillance of severe acute respiratory illness (SARI) in children and adults at the Queen Elizabeth Central Hospital (QECH), Blantyre (pediatric surveillance, adult surveillance, use of Taqman Array Card to characterize severe acute respiratory infection in children under 5 years (TAC), estimating the economic burden of acute respiratory illness in pregnant women and infants in Malawi: a cohort study (ARICOST), and infant burden; Supplementary Table 1 and Supplementary Figure 1) [24, 25]. This government hospital provides free healthcare to an urban and periurban population of approximately 1.3 million across Blantyre District. We also enrolled adults in a prospective observational study that evaluated the association between human immunodeficiency virus (HIV) and influenza infection in Malawian adults (BASH-FLU) between 2013 and 2015 [26]. Asymptomatic adults were enrolled from BASH-FLU cohort participants attending a routine study visit, while asymptomatic children were enrolled from outpatient and vaccination clinics (TAC) between June 2014 and August 2015 (Supplementary Table 1).

### Case and Control Definitions

SARI was defined as a physician-diagnosed acute lower respiratory infection requiring hospitalization in a child aged 8 days to 2 months; or an acute respiratory infection with reported/documented fever (≥38°C) and cough, requiring hospital admission for persons aged ≥2 months. Influenza-like illness (ILI) was defined as reported/documented fever (≥38°C) and ≥2 of the following symptoms: cough, rhinorrhea, sore throat, myalgia, headache, and vomiting/diarrhea [26].

Asymptomatic adult controls were participants enrolled in the BASH-FLU cohort study who attended a routine study visit [26]. Two asymptomatic pediatric control groups were recruited: (1) healthy children who attended the health facility for immunization; and (2) children from outpatient clinics who reported no fever, respiratory symptoms, or other signs of infection, who had not been admitted in the preceding 14 days or had a SARI episode in the previous 30 days.

### Patient Recruitment and Data Collection

For sentinel SARI surveillance, recruitment targets were set to ensure representativeness by epidemiological week (Supplementary Table 2). Pediatric controls were recruited at a ratio of approximately 2 cases to 1 control from outpatient settings, frequency-matched for age (1–5, 6–11, 12–23, 24–59 months) and time (by week).

For the BASH-FLU study, adults aged ≥18 years enrolled in a prospective cohort to evaluate the impact of HIV infection on the incidence of influenza infections attended the study clinic every 2 months (asymptomatic control specimens) and were also instructed to attend when experiencing an ILI [26]. Thus BASH-FLU participants contributed both asymptomatic and ILI samples.

Standardized case report forms (Teleform, 2011–2017 and Open Data Kit, 2017) were used to capture demographic and clinical data. This analysis included all samples obtained from participants of the SARI surveillance studies and BASH-FLU cohort study that were tested for HCoVs.

### Specimen Collection and Laboratory Procedures

The collection and processing of respiratory specimens has been described previously [24, 25]. In brief, nasopharyngeal aspirates (sentinel surveillance 2011–2014) and paired oropharyngeal/nasopharyngeal swabs (FLOQSwabs, Copan Diagnostics, Brescia, Italy) (BASH-FLU study 2013–2015; sentinel surveillance 2015–2017) were collected from participants and stored in universal transport medium at −80°C. These were batch tested for respiratory viruses including HCoVs OC43, NL63, 229E, and HKU1 using the FTD respiratory pathogens 33 kit (Fast-track Diagnostics Ltd, Luxembourg). Between May 2014 and July 2015, specimens collected from children aged 8 days to 59 months who presented to QECH with SARI as well as age- and time-frequency matched controls were tested for respiratory pathogens using the TaqMan array card (Life Technologies, Carlsbad, California) [27].

The methods of the FTD testing have been previously described [25], and results with the TaqMan array card have been found to be comparable [28]. Similar procedures were used for FTD and TaqMan array card. In summary, total nucleic acids were extracted using standard protocols from 300-μL aliquots of each specimen with the Qiagen BioRobot Universal System using the QIAamp One-For-All nucleic acid kit (Qiagen Ltd, Manchester, United Kingdom). The quantity of nucleic acid used per reaction was 5 μL for the TaqMan array card real-time reverse-transcription polymerase chain reaction (RT-PCR) to test for 30 pathogens simultaneously (Supplementary Table 3). Molecular testing was done using the AG-Path-ID One-Step RT-PCR 2× buffer and 25× enzyme mix. The specimens were analyzed using the ViiATM 7 Real-Time PCR machine.

### Statistical Analysis

Statistical analyses were performed using R version 4.1.1 software. We conducted multivariable analysis of factors associated with the positive test rate (number of positive tests per week) for each HCoV using negative binomial models (to account for overdispersion) with an offset of log number of tests. Samples that tested positive for non-HCoV viruses were excluded. Individual-level test results were aggregated by week into strata of participant age, sex, illness severity (ILI, SARI, or asymptomatic), and HIV status. We sought to model the average seasonal pattern across all observed years by including variables representing first and second order harmonics of week of year (w), to capture multiple seasonal peaks if present: $\beta_{1}\sin\left(\frac{2w\pi }{52}\right)+\beta_{2}\cos\left(\frac{2w\pi }{52}\right)+\beta_{3}\sin\left(\frac{4w\pi }{52}\right)+\beta_{4}\cos\left(\frac{4w\pi }{52}\right)$ [29].

The inclusion of third-order harmonics was explored for each HCoV, and likelihood ratio tests were used to determine their inclusion: this was warranted for OC43 and NL63 only. Last, we included a linear term for week since the start of the observation period to identify any long-term secular trends. Incidence rate ratios (IRRs) and 95% confidence intervals (CIs) were estimated for variables from the fitted negative binomial models. Seasonality was assessed and visualized for HCoV-positive ILI and SARI specimens.

The pathogen-attributable fraction (PAF) was calculated for each HCoV in pediatric and adult specimens, to estimate the proportion of HCoVs detected that was attributable to illness (ILI or SARI) [30]. For both groups, cases (ILI and SARI) were restricted to match the recruitment period of asymptomatic controls. PAFs were calculated using adjusted odds ratios (aORs; calculated as odds of ILI or SARI predicted by each HCoV, adjusted for sex, season, HIV status, and detection of 1 or more other respiratory viruses in adults, and for the same variables except HIV status in children) as 100 × $\frac{aOR-1}{aOR}$ and reported with 95% CIs (based on aOR 95% CIs).

### Ethical Considerations

Ethical approval for all studies was obtained through the University of Malawi College of Medicine Research Ethics Committee (COMREC), the Malawi National Health Science Research Committee, and other ethical committees where necessary (Supplementary Table 4). For studies funded by the US Centers for Disease Control and Prevention (CDC), the CDC institutional review board relied on ethical approval through COMREC.

## RESULTS

### Participant Demographics

We included 6107 specimens from 5802 individuals tested for seasonal coronaviruses between 2011 and 2017. BASH-FLU cohort study participants contributed control and ILI samples. Most specimens were obtained from individuals with SARI (n = 5045), followed by asymptomatic controls (n = 717) and those with ILI (n = 345) (Supplementary Table 5). Sex distribution was even (50.9% of specimens from males). More than a third of specimens were from infants <1 year of age (39.6% [n = 2419]), while 44.1% (n = 2695) were from adults (aged ≥15 years). Among those with known HIV status (n = 4888), 37.0% (n = 1809) were HIV infected. HIV prevalence was substantially higher among adults (60.6% [1621/2673]) than children (8.5% [188/2211]). Of note, the national HIV prevalence was 8.8%–10.5% (among adults aged 15–49 years) during the study period [31].

### Prevalence of Positive HCoV PCR Tests Among SARI and ILI Patients and Among Controls

Overall, 539 of 6107 (8.8%) specimens were positive for at least 1 HCoV. Twelve pediatric and 14 adult specimens had >1 HCoV detected; all were from individuals with SARI. Most of these specimens (19/26) were positive for 2 HCoVs, 3 were positive for 3 HCoVs, and 4 were positive for all 4 HCoVs. Among adult specimens with multiple HCoVs, most (10/14) were obtained from HIV-infected individuals.

OC43 was the most frequently detected HCoV (3.1% [191/ 6107]), significantly more frequent than NL63 (2.5% [153/ 6107]; *P* < .05), HKU1 (2.0% [121/6091]; *P* < .001), or 229E (1.9% [113/6107]; *P* < .001) (Table 1). HCoVs were more frequently detected in the hot, dry (12.1% [189/1561]), and hot, wet seasons (10.5% [172/1640]), compared to the cool, dry season (4.4% [129/2910]). Overall, 505 HCoVs were detected in specimens collected from 466 of 5045 patients with SARI, with OC43 being the most prevalent (3.6% [182/5045]). NL63 was most frequently identified HCoV in both patients with ILI (7.0% [24/345]) and asymptomatic controls (1.7% [12/717]).

Among 3416 pediatric specimens, 280 (8.2%) were positive for 1 or more HCoVs. The most frequently identified HCoV in children was OC43 (n = 124 [3.6%]), while 229E was the least frequently detected (n = 36 [1.1%]). The prevalence of individual HCoV types was not statistically different among SARI cases compared to asymptomatic controls.

Of 2691 adult specimens, 277 (10.3%) were positive for 1 or more HCoVs; the majority (78.7% [218/277]) of HCoVs were collected from patients with SARI, followed by those with ILI (16.2% [45/277]) and asymptomatic controls (5.1% [14/277]) (Table 1). All 4 HCoVs had similar prevalence among adults (2.2%–2.9%; Table 1).

In terms of age distribution, the median age of all specimens was 3.0 years. Median age was highest among those positive for 229E (26.5 years [interquartile range {IQR}, 2.0–35.7 years]), while median age for OC43-positive samples was lowest at 2.0 years (IQR, 1.0–29.2 years) (Supplementary Figure 2).

### Positive Test Rate of HCoVs in Patients With ILI and SARI

**Figure 1.**
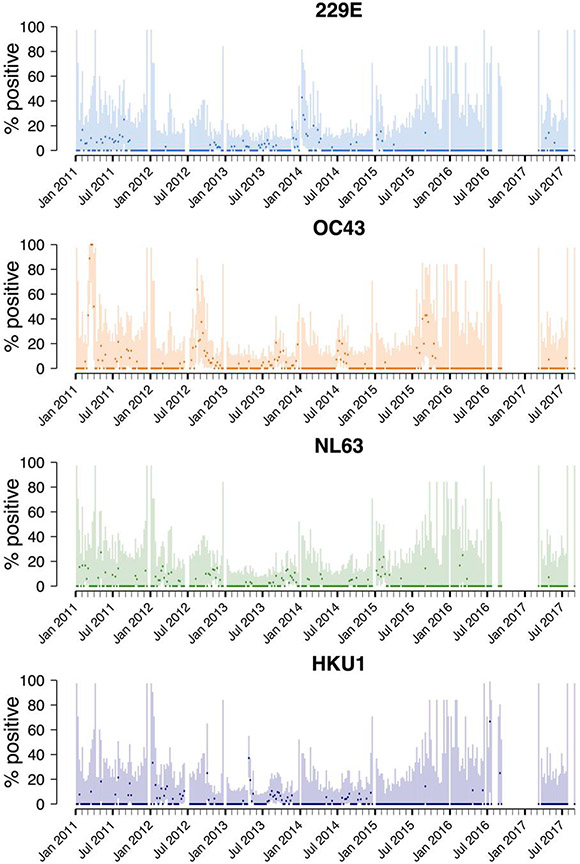
Percentage polymerase chain reaction positivity for human seasonal coronaviruses (229E, OC43, NL63, and HKU1) in individuals with severe acute respiratory illness and influenza-like illness in Blantyre, Malawi, 2011–2017. Lines show 95% binomial confidence intervals.

**Figure 2.**
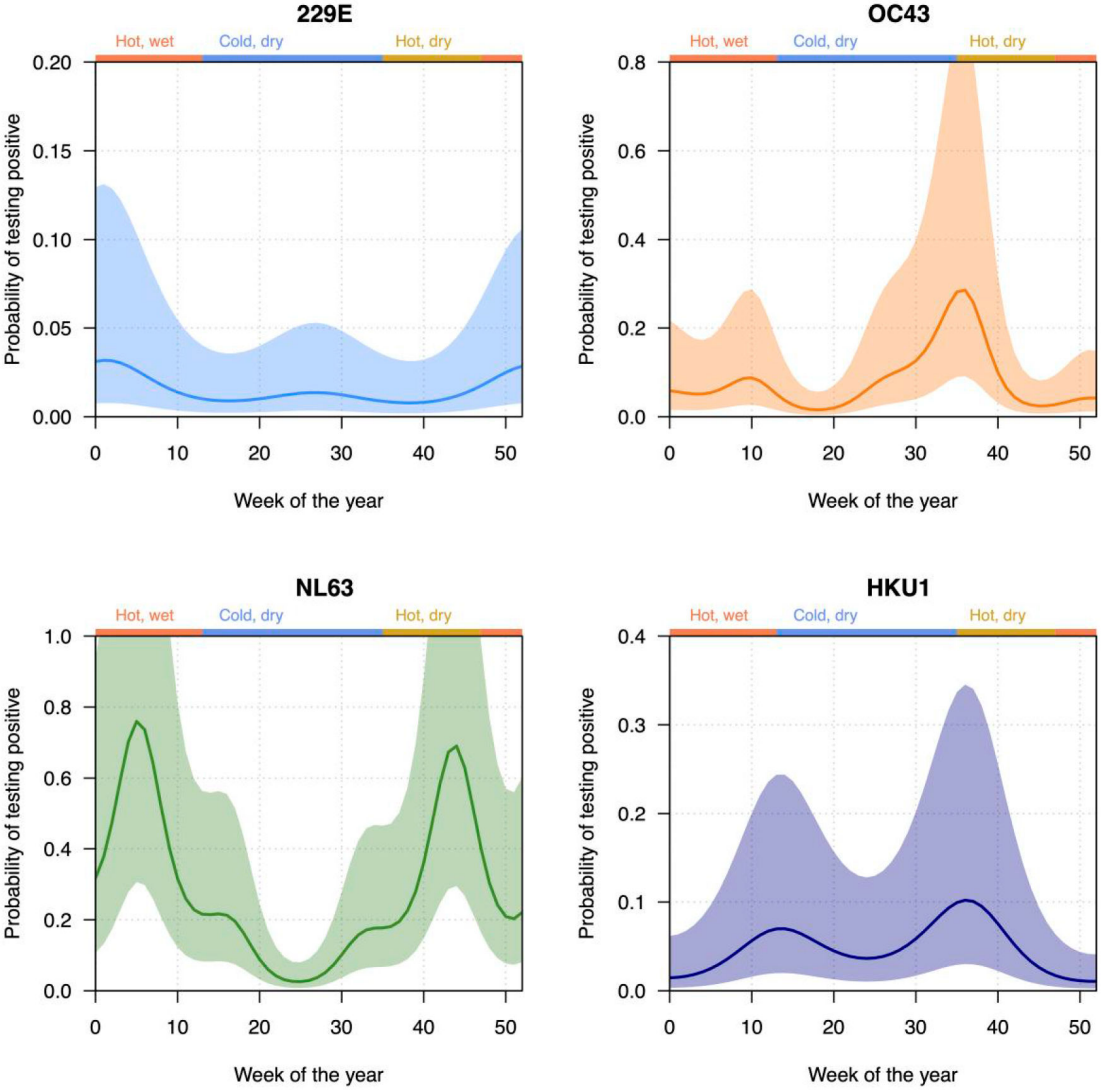
Probability of testing positive for each individual human seasonal coronavirus (229E, OC43, NL63, and HKU1) over time (given age >1 year, male sex, negative human immunodeficiency virus status, and influenza-like illness). Seasons are shown on the top of the figure: hot, wet; cold, dry; and hot, dry. Solid line indicates mean estimate, while the shaded area shows the 95% confidence interval.

Restricting to samples that did not test positive for other respiratory viruses, after adjusting for age, sex, HIV status, clinical severity, and temporal patterns, the weekly test positivity of OC43 was significantly higher in younger age groups (<1 year: adjusted IRR [aIRR], 3.11 [95% CI, 1.66–5.81]; 1–14 years: aIRR, 2.22 [95% CI, 1.13–4.35]), compared to 15–39 years (Table 2). Compared to asymptomatic controls, NL63 was more frequently detected in individuals with ILI (aIRR, 9.60 [95% CI, 3.25–28.30]), while 229E was more frequently detected in SARI (aIRR, 8.99 [95% CI, 1.81–44.70]) compared to asymptomatic controls. We found no significant association between sex or HIV status and positive test rate of any of the HCoVs, while all 4 HCoVs demonstrated seasonal patterns (Table 2, Figures 1 and 2). We found a slight temporal decline in test positivity of OC43, NL63, and HKU1 over time.

### Seasonality

An outbreak of OC43 occurred in March 2011, when 100% (13/13) of adult and 67.7% (21/31) of pediatric specimens were OC43 PCR positive, followed by a spike in cases in August 2012, when 27% (10/37) of pediatric and 28.6% (6/21) of adult specimens were PCR positive (Figure 1, Supplementary Figure 3). Additionally, the prevalence of OC43 cases was increased in children in September 2013 (10.0% positive [7/70]), July 2014 (25.8% [16/62]), and September 2015 (27% [7/26]). There was a spike of 229E cases in January 2014, when 25.6% (10/39) of adult specimens were PCR positive.

The seasonality of HCoVs was assessed by predicting the proportion of specimens testing positive for each species over time (week of the year) for a standardized demographic (infants (<1 year), male sex, HIV uninfected, and ILI), using the fitted negative binomial models. All HCoVs showed biannual peaks, with more complicated seasonality patterns being present in OC43 and NL63 (Table 2, Figure 2). 229E had the largest peak between week 50 and week 5, while HKU1 had equivalent-sized peaks between weeks 10–20 and 30–40. OC43 had a dominant peak between weeks 30 and 40, and NL63 had 2 peaks between weeks 0–10 and 40–50.

### Codetection With Other Respiratory Viruses

Two or more respiratory viruses were detected in 245 of 5045 (4.9%) SARI cases, 6 of 345 (1.7%) ILI cases, and 9 of 717 (1.3%) asymptomatic controls. Among the 539 specimens that were positive for 1 or more HCoVs, 247 (45.8%) had another respiratory virus present (ie, 13 samples were coinfected with multiple HCoV species only). Codetection of HCoV and another respiratory virus was significantly more common in SARI (4.6% [232/5045]), compared to ILI (1.7% [6/345]; *P* = .012) and asymptomatic controls (1.3% [9/717]; *P* < .01) (Supplementary Figure 4). Codetection of HCoV and another respiratory virus was also more common in children than adults (4.5% [154/3420] vs 3.5% [93/2691]; *P* = .046). In children, rhinovirus was most commonly codetected with 229E, NL63, and HKU1, and bocavirus with OC43 (Supplementary Figure 5A). Among adults, bocavirus was most frequently codetected with 229E, rhinovirus with OC43, adenovirus and bocavirus with NL63, and adenovirus with HKU1 (Supplementary Figure 5B).

### Pathogen-Attributable Fractions

Specimens from 474 asymptomatic adult controls were obtained between April 2013 and April 2015; 345 ILI and 1094 SARI cases were enrolled during this period (Supplementary Table 6). NL63 (PAF, 85.1% [95% CI, 64.8%–94.6%]) was significantly associated with ILI, while 229E (PAF, 86.5% [95% CI, 61.2%–96.8%]) and OC43 (PAF, 89.4% [95% CI, 47.1%–99.4%]) were significantly associated with SARI (Table 3).

Asymptomatic pediatric controls (n = 241) were recruited between June 2014 and August 2015 (Supplementary Table 6), along with 714 SARI cases. None of the HCoVs were associated with pediatric SARI.

## DISCUSSION

We characterized the epidemiology and circulation patterns of seasonal HCoVs over 7 years before the COVID-19 pandemic. Overall, OC43 was the most prevalent HCoV in children, while 229E was more frequently detected in adults. NL63 was associated with ILI, while 229E was associated with SARI. By comparing to contemporaneous specimens from asymptomatic controls, we ascertained that 229E and OC43 had a high PAF for SARI in adults, while NL63 had a high PAF in adult ILI. This means that the majority of ILI and SARI that were PCR positive for these HCoVs were the likely cause of their illness. In contrast, the prevalence of all HCoVs was similar between cases and controls in children. All 4 HCoVs peaked twice yearly, though each had distinct seasonality. Codetection with another respiratory virus, predominantly rhinovirus, was more frequent in SARI cases and in pediatric cases than in adult specimens.

Few studies have evaluated the epidemiology and seasonal patterns of HCoVs in Africa. Most have small sample sizes; only 4 of 12 studies included >1000 participants [19, 20, 23, 32]. All but 3 included children only [21, 22]. Most encompassed short time periods, with only 5 studies spanning over 2 years [20, 22, 23, 32, 33]. Inclusion of multiple years of data is useful to account for annual fluctuations. Four studies included control subjects [16, 21, 32, 34], though 3 had <100 controls [16, 18, 34], thus precluding meaningful case-control comparison and estimation of the attribution of virus detection to clinical disease. Moreover, Berkley et al [16] did not recruit control subjects at the same time as cases, while Owusu et al [21] focused recruitment during certain seasons.

In temperate settings, HCoVs predominantly circulate in winter months [2, 3, 35, 36], except for China where HCoVs are less seasonal [15]. Two recent systematic reviews on HCoV seasonality highlighted an underrepresentation of African studies [15, 37]. A study in Kenyan children found no seasonal patterns [20]. A study from Ghana found higher circulation of HCoVs in the harmattan (season between the end of November and mid-March, characterised by dry and dusty wind from the Sahara over West Africa) and wet seasons, but the seasonality of individual HCoVs was not evaluated [21]. Last, a Senegalese surveillance study identified seasonal peaks for OC43 (November–January) and NL63 (September–January) but not for 229E and HKU1 [23]. However, controls were not included. In contrast, our study encompassed a 7-year period and included all ages with varying illness severity, as well as asymptomatic controls. We restricted our case-control analysis to time periods where both cases and controls were recruited. In contrast to temperate settings, all HCoVs had biannual peaks though individual HCoVs had distinct seasonality; both betacoronaviruses, OC43 and HKU1, had significant peaks in August–September and a smaller peak in February–March; alphacoronavirus 229E, June–July and December–January; and NL63, January–February and October–November (Figure 2). Circulatory peaks of individual HCoVs did not correlate with climactic conditions. Of note, data from the sentinel SARI surveillance at QECH have also shown annual cycles but variable peaks in the circulation of respiratory syncytial virus and influenza viruses [24, 25].

Among adults, 229E and OC43 were associated with SARI, while NL63 was associated with ILI. In contrast, there was no association between any HCoVs with SARI in children. The few studies that have evaluated the contribution of HCoVs to mild and severe respiratory illness have yielded conflicting results. Similar to our findings, a case-control study of older children and adults from Ghana found that 229E and OC43 were associated with upper respiratory tract infections [38], while a birth cohort in South Africa also found that HCoVs (particularly OC43) were associated with lower respiratory tract infections in infants [33]. Conversely, a study in South African and Zambian children found that HCoVs were more common in asymptomatic controls compared to children aged <5 years hospitalized with clinical pneumonia [32]. A case-control study in Australia also concluded that HCoVs did not contribute to pneumonia in children [39]. These studies highlight that a virus detected in the upper respiratory tract does not necessarily imply a causative pathogen. Asymptomatic carriage of certain viruses is common, so the inclusion of controls is critical. The contrasting findings between studies may be due to varying case and control definitions and may also be partly due to uncontrolled confounding, such as coinfections with other respiratory pathogens, which occur frequently [40, 41] and have been linked to increased disease severity [42]. Here, codetection of HCoVs and other respiratory viruses was significantly more frequent in children than adults, which has been previously reported [23, 43].

Our study had several limitations. It was focused on 1 urban setting in Malawi, which may not be representative of the country’s population. We did not have data on HCoVs in pediatric ILI; further studies are needed to explore this. Controls were only enrolled between 2013–2015, and fewer were recruited compared to cases, particularly among children; hence, the PAF estimates had wide CIs. When assessing PAF, we were unable to explore interactions (eg, between HCoV positivity and sex or HIV status) due to the limited specimen size. As bocavirus is not included in the TaqMan array card, bocavirus results were missing for 721 children enrolled in the TAC study. The modeling analysis did not account for repeated testing from the same individual, or for different timings of infection seasons between years. Finally, although we included 7 years of data, any stochastic large outbreak would feed into seasonality pattern and could be a strong influence of observed seasonality.

Against the backdrop of scarce data on HCoV epidemiology in Africa, we have provided a detailed description of the contribution of seasonal coronaviruses to mild and severe acute respiratory presentations as well as asymptomatic infection in children and adults in southern Malawi before the COVID-19 pandemic. Additionally, we demonstrated that each HCoV species had distinct seasonality, which differed from that reported in temperate settings. These data are useful for several reasons. First, future waves of SARS-CoV-2 may mirror that of endemic human betacoronaviruses (OC43 and HKU1). The identification of seasonal epidemic patterns, albeit different to that in temperate settings, may provide insight into future SARS-CoV-2 seasonality. Second, we can begin to examine whether cross-protection from circulating coronaviruses plays a role in milder COVID-19 observed in Africans. Ongoing surveillance of the circulation of HCoVs, in addition to other respiratory viruses including SARS-CoV-2, will be key to understand how they interact with one another. The impact of HCoVs in high-risk individuals, such as those with HIV infection or malnutrition, requires further study.

## Supplementary Material

supplement

## Figures and Tables

**Table 1.** Prevalence of Seasonal Coronavirus Polymerase Chain Reaction Positivity in Specimens Obtained From Individuals With Severe Acute Respiratory Illness, Influenza-like Illness, and Asymptomatic Controls in Blantyre, Malawi, 2011–2017

	Pediatric (Age <15 y)						Adult (Age ≥15 y)					
Characteristic	No. of Specimens Tested	≥1 HCoV Detected^a^	229E	OC43	NL63	HKU1^a^	No. of Specimens Tested	≥1 HCoV Detected	229E	OC43	NL63	HKU1^b^
Total	3416	280 (8.2)	36 (1.1)	124 (3.6)	79 (2.3)	62 (1.8)	2691	277 (10.3)	77 (2.9)	67 (2.5)	74 (2.7)	59 (2.2)
Sex												
Male	1847	185 (10.0)	22 (1.2)	76 (4.1)	44 (2.4)	43 (2.3)	1266	118 (9.3)	41 (3.2)	24 (1.9)	29 (2.3)	24 (1.9)
Female	1573	116 (7.4)	14 (0.9)	48 (3.1)	35 (2.2)	19 (1.2)	1425	159 (11.2)	36 (2.5)	43 (3.0)	45 (3.6)	35 (2.5)
Age, y												
<1	2416	196 (8.1)	25 (1.0)	82 (3.4)	55 (2.3)	34 (1.4)	…	…	…	…	…	…
1–14	1000	115 (11.5)	11 (1.1)	42 (4.2)	24 (2.4)	28 (2.8)	…	…	…	…	…	…
15–39	…	…	…	…	…	…	1872	188 (10.0)	53 (2.8)	45 (2.4)	49 (2.6)	41 (2.2)
≥40	…	…	…	…	…	…	819	89 (10.9)	24 (2.9)	22 (2.7)	25 (3.1)	18 (2.2)
HIV status												
Negative	2023	202 (10.0)	22 (1.1)	91 (4.5)	47 (2.3)	42 (2.1)	1052	102 (9.7)	29 (2.8)	21 (2.0)	26 (2.5)	26 (2.5)
Positive	188	21 (11.2)	2 (1.1)	13 (6.9)	3 (1.6)	3 (1.6)	1621	170 (10.5)	48 (2.9)	46 (2.8)	46 (2.8)	30 (1.9)
Missing	1205	78 (6.5)	12 (1.0)	20 (1.7)	29 (2.4)	17 (1.4)	18	5 (27.7)	0 (0)	0 (0)	2 (11.1)	3 (16.6)
Clinical severity												
SARI	3175	287 (9.0)	33 (1.0)	121 (3.8)	73 (2.3)	60 (1.9)	1870	218 (11.7)	66 (3.5)	61 (3.3)	44 (2.3)	47 (2.5)
ILI^c^	…	…	…	…	…	…	345	45 (13.0)	8 (2.3)	5 (1.5)	24 (7.0)	8 (2.3)
Asymptomatic control	241	14 (5.8)	3 (1.2)	3 (1.2)	6 (2.5)	2 (0.8)	476	14 (2.9)	3 (0.6)	1 (0.2)	6 (1.3)	4 (0.8)
Season												
Hot, wet (Dec–Mar)	852	70 (8.2)	11 (1.3)	20 (2.3)	33 (3.9)	6 (0.7)	785	101 (12.9)	32 (4.1)	25 (3.2)	29 (3.7)	15 (1.9)
Cool, dry (Apr–Aug)	1709	129 (7.5)	17 (1.0)	63 (3.7)	13 (0.8)	36 (2.1)	1200	89 (7.4)	26 (2.2)	21 (1.8)	13 (1.1)	29 (2.4)
Hot, dry (Sep–Nov)	855	102 (11.9)	8 (0.9)	41 (4.8)	33 (3.9)	20 (2.3)	706	87 (12.3)	19 (2.7)	21 (3.0)	32 (4.5)	15 (2.1)

Data are presented as No. (%) unless otherwise indicated.

Abbreviations: HCoV, human coronavirus; HIV, human immunodeficiency virus; ILI, influenza-like illness; SARI, severe acute respiratory illness.

a HKU1 results were missing for 16 pediatric samples; denominator for the proportions was adjusted accordingly.

b HKU1 results were missing for 13 adult samples; denominator for the proportions was adjusted accordingly.

c No data were collected on pediatric ILI.

**Table 2.** Multivariable Analysis of Factors Associated With Positive Human Coronavirus 229E, OC43, NL63, and HKU1 Polymerase Chain Reaction Among Those With Influenza-like Illness and Severe Acute Respiratory Illness, Compared to Healthy Controls in Blantyre, Malawi, 2011–2017

	229E (n = 56)		OC43 (n = 92)		NL63 (n = 74)		HKU1 (n = 63)	
Characteristic	IRR^a^	(95% CI)	IRR^a^	(95% CI)	IRR^a^	(95% CI)	IRR^a^	(95% CI)
Sex								
Male	…	…	…	…	…	…	…	…
Female	1.01	(.52–1.93)	1.18	(.77–1.80)	1.21	(.75–1.95)	0.92	(.53–1.59)
HIV status								
Negative	…	…	…	…	…	…	…	…
Positive	0.61	(.30–1.26)	1.48	(.87–2.55)	0.80	(.46–1.37)	0.65	(.34–1.24)
Missing	NA	NA	NA	NA	1.53	(.43–5.45)	2.21	(.57–8.59)
Clinical severity								
Asymptomatic	…	…	…	…	…	…	…	…
ILI	5.35	(.87–32.80)	7.21	(.84–62.1)	**9.60**	**(3.25–28.30)**	2.75	(.75–9.99)
SARI	**8.99**	**(1.81–44.70)**	7.17	(.97–53.2)	1.35	(.44–4.14)	1.59	(.51–4.92)
Seasonality^b^								
Sine (first)	0.98	(.58–1.66)	0.59	(.42–.83)	0.73	(.49–1.09)	0.76	(.52–1.10)
Cosine (first)	**1.83**	**(1.19–2.83)**	0.75	(.48–1.17)	4.64	**(2.55–8.44)**	0.48	(.28–.85)
Sine (second)	0.92	(.59–1.42)	**2.16**	**(1.49–3.13)**	1.01	(.69–1.47)	1.22	(.82–1.83)
Cosine (second)	**2.28**	**(1.34–3.87)**	0.68	(.44–1.04)	**0.44**	**(.25–.78)**	**0.50**	**(.31–.81)**
Sine (third)^c^	…	…	0.89	(.64–1.24)	1.33	(.92–1.92)	…	…
Cosine (third)^c^	…	…	1.13	(.81–1.59)	0.66	(.43–1.01)	…	…
Secular time, wk^d^	1.00	(1.00–1.01)	**0.99**	**(.99–1.00)**	**0.99**	**(.99–1.00)**	**0.99**	**(.99–1.00)**
Age group, y^e^								
<1	…	…	**3.11**	**(1.66–5.81)**	…	…	…	…
1–14	0.37	(.07–1.89)	**2.22**	**(1.13–4.35)**	0.49	(.19–1.32)	**2.66**	**(1.03–6.89)**
15–39	1.75	(.64–4.78)	…	…	0.70	(.33–1.51)	2.10	(.84–5.21)
≥40	1.9	(.67–5.78)	0.94	(.46–1.90)	0.60	(.25–1.42)	1.30	(.43–3.90)

Samples that tested positive for respiratory viruses other than human coronaviruses were excluded from this analysis. Variables significant at *P* < .05 are in bold.

Abbreviations: CI, confidence interval; HIV, human immunodeficiency virus; ILI, influenza-like illness; IRR, incidence rate ratio; SARI, severe acute respiratory illness.

a Adjusted for all listed variables.

b See Figure 2 for details on seasonality.

c Third harmonics were only included for OC43 and NL63 models (based on fit).

d Upper bound of CI is <1.00.

e The reference age group for OC43 was changed to 15–39 years for easier interpretation of IRRs >1.

**Table 3.** Odds Ratios and Pathogen-Attributable Fractions Associated With Human Coronaviruses in Severe Acute Respiratory Illness and Influenza-like Illness in Pediatric and Adult Patients, 2013–2015

	ILI					SARI				
HCoV	Observed Prevalence, %	Univariable OR (95% CI)	Multivariable OR^a^ (95% CI)	PAF^b^, % (95% CI)	Adjusted Prevalence, %	Observed Prevalence, %	Univariable OR (95% CI)	Multivariable ORa (95% CI)	PAF^b^, % (95% CI)	Adjusted Prevalence, %
Pediatric (June 2014–August 2015)										
229E	…	…	…	…	…	1.1	0.90 (.03–4.13)	1.39 1.36–6.81)	28.1 (0–85.3)	…
OC43	…	…	…	…	…	3.1	2.52 (.86–10.70)	2.62 (.84–11.60)	61.8 (0–91.4)	…
NL63	…	…	…	…	…	2.1	0.84 (.34–2.38)	0.95 (.35–2.88)	…	…
HKU1	…	…	…	…	…	0.8^c^	NA	NA	NA	…
Adult (April 2013–April 2015)										
229E	0.7	**3.74 (1.07–17.20)**	3.61 (1.00–17.00)	72.3 (0–94.1)	…	3.7	**5.98 (2.16–24.80)**	**7.42 (2.58–31.40)**	**86.5 (61.2–96.8)**	3.2
OC43	0.5	NA	NA	NA	…	1.8	**8.85 (1.84–159.00)**	**9.41 (1.89–171.00)**	**89.4 (47.1–99.4)**	1.6
NL63	2.2	**5.86 (2.52–16.00)**	**6.70 (2.84–18.50)**	**85.1 (64.8–94.6)**	1.9	1.1	0.87 (.34–2.51)	0.67 (.24–2.02)	…	…
HKU1	0.7^c^	2.80 (.87–10.60)	2.67 (.82–10.20)	62.5 (0–90.2)	…	1.8	2.20 (.83–7.59)	2.06 (.75–7.30)	51.5 (0–86.3)	

Values in bold indicate significant results. Abbreviations: CI, confidence interval; HCoV, human coronavirus; ILI, influenza-like illness; NA, not applicable; OR, odds ratio; PAF, pathogen-attributable fraction; SARI, severe acute respiratory illness.

a Adjusted for sex, season, human immunodeficiency virus status (adults only), and detection of another respiratory virus (polymerase chain reaction positive for 1 or more of adenovirus, bocavirus, human metapneumovirus, parainfluenza virus 1–4, influenza A and B, rhinovirus, and respiratory syncytial virus).

b Only calculated for those with OR >1.

c Fewer than 10 positive samples; hence, ORs and PAF are omitted.
