# Supplementary material for: Epidemiology of Human Seasonal Coronaviruses Among People With Mild and Severe Acute Respiratory Illness in Blantyre, Malawi, 2011–2017
Source: J Infect Dis. 2024 Feb 14;230(2):e363–73. doi: 10.1093/infdis/jiad587 (PMC11322416; doi:10.1093/infdis/jiad587)
Supplement: jiad587_Supplementary_Data [file jiad587_supplementary_data.docx]

**Supplementary material**

[Supplementary Figures 2](#_Toc155690416)

[Supplementary Figure 1. Timeline, age categories and type of HCoV testing of included paediatric and adult studies and surveillance data 2](#_Toc155690417)

[Supplementary Figure 2. Age distribution of all specimens taken and specimens positive for individual seasonal coronaviruses, Blantyre, Malawi, 2011–2017 3](#_Toc155690418)

[Supplementary Figure 3*.* Number of specimens taken per month and number of specimens positive for each HCoV species over time among children and adults with ILI and SARI, Blantyre, Malawi (2011-2017) 4](#_Toc155690419)

[Supplementary Figure 4. Number of specimens positive for another respiratory virus among specimens positive for 229E, OC43, NL63 and HKU1 in A) asymptomatic controls, B) ILI, and C) SARI 6](#_Toc155690420)

[Supplementary Figure 5. Number of specimens positive for another respiratory virus among specimens positive for 229E, OC43, NL63 and HKU1 in A) children and B) adults 7](#_Toc155690421)

[Supplementary Tables 8](#_Toc155690422)

[Supplementary Table 1. Summary details of studies and surveillance platforms from which cases and controls were enrolled 8](#_Toc155690423)

[Supplementary Table 2. Targets for enrolment by specific respiratory pathogen surveillance studies 11](#_Toc155690424)

[Supplementary Table 3. Comparison of pathogens included in the TaqMan array card and FTD respiratory pathogens kit 12](#_Toc155690425)

[Supplementary Table 4. Ethical approvals for included studies 14](#_Toc155690426)

[Supplementary Table 5. Demographics of adult and paediatric participants who provided one or more specimens by disease severity, Queen Elizabeth Central Hospital, Blantyre, Malawi, 2011–2017 (N=6107) 15](#_Toc155690427)

[Supplementary Table 6. Characteristics of participants with SARI, ILI, and asymptomatic control participants included in pathogen-attributable fraction analysis 17](#_Toc155690428)

# **Supplementary Figures**

**Supplementary Figure 1**. Timeline, age categories and type of HCoV testing of included paediatric and adult studies and surveillance data

* Denotes studies that recruited asymptomatic controls.


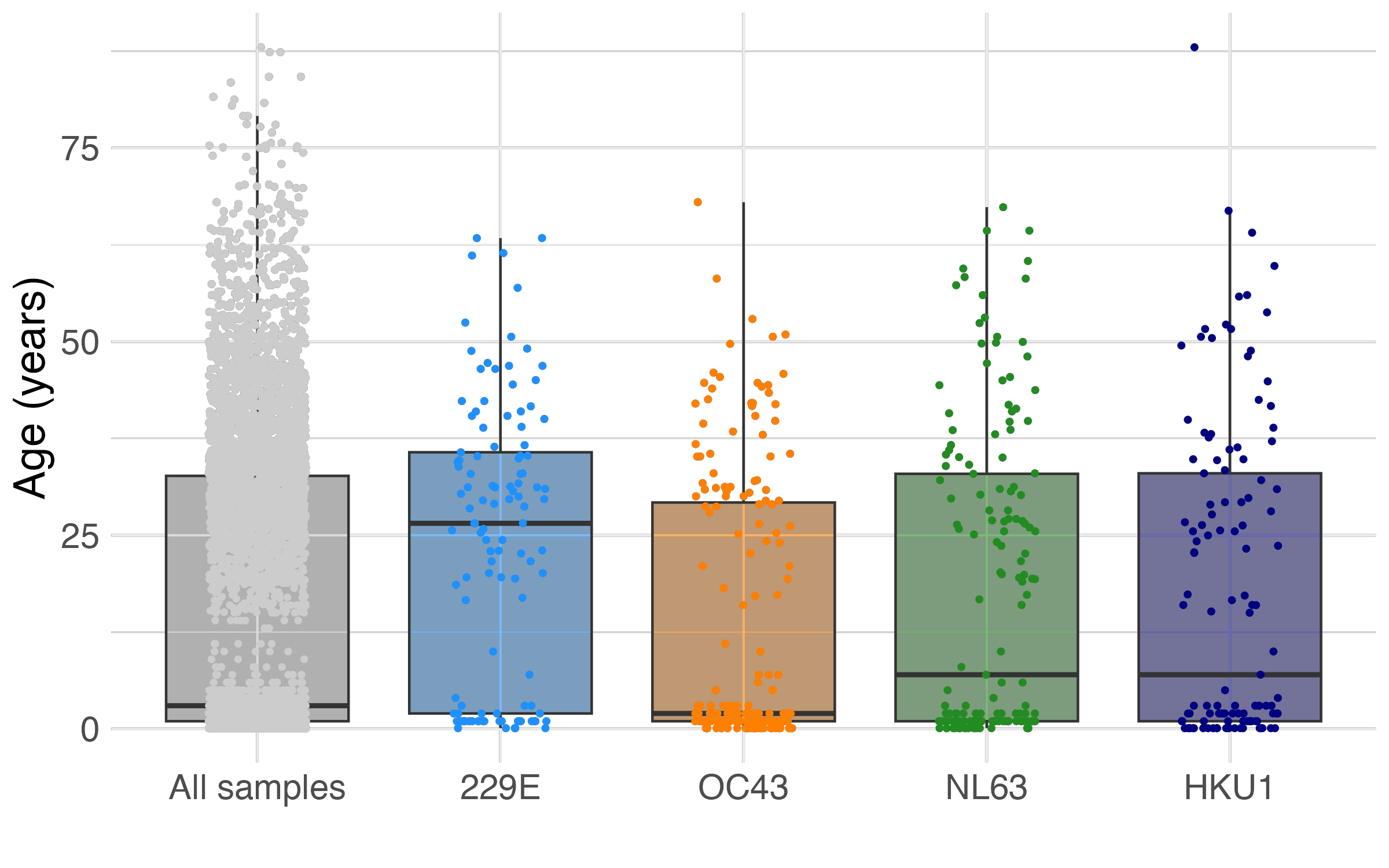


**Supplementary Figure 2.** Age distribution of all specimens taken and specimens positive for individual seasonal coronaviruses, Blantyre, Malawi, 2011–2017

Box plots show median (thick horizontal line and 25^th^ and 75^th^ percentiles (hinges). Whiskers represent 1.5 × interquartile range; dots show outliers.

| **Supplementary Figure 3*.*** Number of specimens taken per month and number of specimens positive for each HCoV species over time among children and adults with ILI and SARI, Blantyre, Malawi (2011-2017) Bars represent number of specimens positive for HCoVs (left y-axis). Black line and black dots show number of specimens taken (right y-axis). |
| --- |

## **Supplementary Figure 4**. Number of specimens positive for another respiratory virus among specimens positive for 229E, OC43, NL63 and HKU1 in **A)** asymptomatic controls, **B)** ILI, and **C)** SARI

Some specimens tested positive for HCoV and >1 other virus.

*Bocavirus results unavailable for 721 children recruited to the TAC study.

RSV, respiratory syncytial virus; HMPV, human metapneumovirus; IAV, influenza A virus; IBV, influenza B virus; HPIV, human parainfluenza virus; N, number of samples.

## **Supplementary Figure 5.** Number of specimens positive for another respiratory virus among specimens positive for 229E, OC43, NL63 and HKU1 in **A)** children and **B)** adults

*Bocavirus results unavailable for 721 children recruited to the TAC study.

RSV, respiratory syncytial virus; HMPV, human metapneumovirus; IAV, influenza A virus; IBV, influenza B virus; HPIV, human parainfluenza virus; N, number of samples.

# **Supplementary Tables**

## **Supplementary Table 1.** Summary details of studies and surveillance platforms from which cases and controls were enrolled

| **Study** | **Paediatric surveillance**^24^ | **TAC** | **ARICOST** | **Infant Burden** | **Adult surveillance**^22^ | **BASH-FLU**^23^ |
| --- | --- | --- | --- | --- | --- | --- |
| **Enrolment** | Cases:  Children aged 3 months to 14 years meeting the SARI definition categorised according to age, primarily: 1) respiratory disease with signs of lower respiratory infection; 2) reported or recorded fever (>38°C); 3) symptom onset < 7 days. For a period of 1 year, ILI cases of the same age were recruited. | Cases:  Children aged <5 years, hospitalised with SARI.    Controls: Outpatient children aged <5 years enrolled within 10 days of a case; not experiencing respiratory symptoms fever, diarrhoea, or other signs of infection and with no history of admission in previous 14 days, or SARI/study enrolment in previous 30 days. | Cases: Infants hospitalised with SARI were enrolled from existing paediatric surveillance studies and further consented for detailed economic questions and a household visit at 6 weeks post discharge. | Cases:  IInfants aged 8 days to 11 months admitted to two hospitals (QECH and Chikwawa district hospital) enrolled by systematic sampling (see Methods). Infants were excluded if admitted for surgery, trauma, hydrocephalus, oncology and infants with major congenital anomalies that might impact immune function will also be excluded. | Cases: Patients presenting to QECH Emergency Department fulfilling SARI criteria: 1) an acute respiratory illness with symptom onset < 7 days; 2) reported or recorded fever (>38°C); 3) cough or sore throat; and 4) shortness of breath or difficulty breathing. | Cases and Controls:  Prospective cohort of 608 adult participants (>18 years; n=360 (59%) HIV-infected) who attended the study clinic with ILI (cases) or for routine visits (asymptomatic controls) |
| **Specimen type** | NPA and NP/OP swabs | NP/OP swabs | NP/OP swabs | NP/OP swabs | NPA | NP/OP swab |
| **Number of specimens included** | 2181 | 721 | 294 | 232 | 1119 | 1560 |
| **Sex – male N (%)** | 1197 (54.9%) | 401 (55.6%) | 107 (36.4%) | 144 (62.1%) | 485 (43.3%) | 778 (49.9%) |
| **Age (years)** |  |  |  |  |  |  |
| <1 | 556 | 201 | 164 | - | - | - |
| 1-4 | 1521 | 515 | 130 | - | - | - |
| 5–14 | 92 | 5 | - | - | - | - |
| 15–39 | 12 | - | - | - | 791 | 1070 |
| >40 | - | - | - | - | 328 | 490 |

NP, nasopharyngeal; OP, oropharyngeal; NPA nasopharyngeal aspirate; QECH, Queen Elizabeth Central Hospital; SARI, severe acute respiratory illness,; N, number.

## **Supplementary Table 2.** Targets for enrolment by specific respiratory pathogen surveillance studies

| Study | Targets* |
| --- | --- |
| TAC study | 10–12 cases and 5–6 controls enrolled per week |
| Paediatric surveillance and Infant Burden | A maximum of 15 paediatric patients, who were eligible according to either the SARI or control definition were recruited to the study per day during set recruitment hours. Recruitment would not exceed the weekly quota. |
| Adult surveillance | A maximum of 15 adult and 10 pregnant women who were eligible according to either the SARI or control definition were recruited to the study per day during set recruitment hours. Recruitment would not exceed the weekly quota. |

SARI, severe acute respiratory illness; TAC, TaqMan Array Card

*If the expected number of cases exceeded the expected number per week, systematic sampling (e.g., every other case, enrolling 2 days on/2 days off, or in 8-hour shifts that rotate on different days) was done to ensure representativeness by epidemiology week. Eligible study participants were recruited and if the daily recruitment cap was not achieved on the day, this would be made up for on the following day.

## **Supplementary Table 3.** Comparison of pathogens included in the TaqMan array card and FTD respiratory pathogens kit

| Pathogen | TaqMan | FTD33 |
| --- | --- | --- |
| Adenovirus - all types | ✓ | ✓ |
| Bocavirus | *X* | ✓ |
| *Bordetella pertussis* | ✓* | ✓ |
| *Chlamydia pneumoniae* | ✓ | ✓ |
| Human Enterovirus | ✓ | ✓ |
| Influenza A | ✓ | ✓ |
| Influenza B | ✓ | ✓ |
| Influenza C | ✓ | ✓ |
| Group A *Streptococcus* | ✓ | *X* |
| *Haemophilus influenzae,* all types | ✓ | ✓ |
| *Haemophilus influenzae* type b | ✓ | ✓ |
| Human Coronavirus (229E) | ✓ | ✓ |
| Human Coronavirus (NL63) | ✓ | ✓ |
| Human Coronavirus (OC43) | ✓ | ✓ |
| Human Coronavirus (HKU1) | ✓ | ✓ |
| Human metapneumovirus | ✓ | ✓ |
| Human parainfluenza virus 1 | ✓ | ✓ |
| Human parainfluenza virus 2 | ✓ | ✓ |
| Human parainfluenza virus 3 | ✓ | ✓ |
| Human parainfluenza virus 4 | ✓ | ✓ |
| *Klebsiella pneumoniae* | ✓ | ✓ |
| *Legionella* species | ✓ | ✓ |
| *Moraxella catarrhalis* | ✓ | ✓ |
| *Mycoplasma pneumoniae* | ✓ | ✓ |
| Parechovirus | *X* | ✓ |
| *Pseudomonas aeruginosa* | ✓ | *X* |
| *Pneumocystis jiroveci* | ✓ | ✓ |
| Human rhinovirus | ✓ | ✓ |
| Respiratory syncytial virus | ✓ | ✓ |
| *Staphylococcus aureus* | ✓ | ✓ |
| *Streptococcus pneumoniae* | ✓ | ✓ |
| *Mycobacterium tuberculosis* | ✓ | *X* |
| *Salmonella spp.* | *X* | ✓ |

*Except *Bordetella parapertussis*

## **Supplementary Table 4.** Ethical approvals for included studies

| Study | COMREC/NHSRC number | Other Institutional approval |
| --- | --- | --- |
| Influenza Surveillance | COMREC P.07/10/958 | Liverpool school of Tropical medicine (RETH000790) |
| BASH-FLU | COMREC P.11/12/1310 | University of Liverpool (Study number 12.43) |
| TAC | COMREC P.10/12/1294 | N/A |
| ARICOST Paediatric | NHSRC #1073 | N/A |
| Infant Burden | COMREC P.08/16/2008 | University of Liverpool (Reference 1553) |

COMREC, University of Malawi College of Medicine Research Ethics Committee; NHSRC, National Health Sciences Research Committee; TAC, TaqMan array card.

## **Supplementary Table 5.** Demographics of adult and paediatric participants who provided one or more specimens by disease severity, Queen Elizabeth Central Hospital, Blantyre, Malawi, 2011–2017 (N=6107)

|  |  | Total number of specimens  N (%)* | SARI  N specimens (%) | ILI**  N specimens (%) | Asymptomatic Controls  N specimens (%) |
| --- | --- | --- | --- | --- | --- |
| Total |  | **6107** | **5045** | **345** | **717** |
| Age group (years) | <1 | 2416 (39.6) | 2258 (44.8) | - | 158 (22.0) |
|  | 1–14 | 1000 (16.4) | 917 (18.2) | - | 83 (11.6) |
|  | 15–39 | 1872 (30.6) | 1326 (26.3) | 230 (66.7) | 317 (44.2) |
|  | ≥40 | 819 (13.4) | 544 (10.8) | 115 (33.3) | 159 (22.2) |
| Sex | Male | 3112 (50.9) | 2669 (52.9) | 133 (38.6) | 310 (43.2) |
|  | Female | 2995 (49.1) | 2376 (47.1) | 212 (61.4) | 407 (56.8) |
| HIV status | Negative | 3075 (50.4) | 2744 (54.4) | 118 (34.2) | 213 (29.7) |
|  | Positive | 1809 (29.6) | 1319 (21.1) | 227 (65.8) | 263 (36.7) |
|  | Unknown | 1223 (20.0) | 982 (19.5) | 0 (0) | 241 (33.6) |
| Year of | 2011 | 543 (8.9) | 543 (10.8) | 0 (0) | 0 (0) |
| recruitment | 2012 | 906 (14.8) | 906 (18.0) | 0 (0) | 0 (0) |
|  | 2013 | 1905 (31.2) | 1576 (31.2) | 144 (41.7) | 185 (25.8) |
|  | 2014 | 1438 (23.5) | 923 (18.3) | 171 (49.6) | 344 (48.0) |
|  | 2015 | 861 (14.1) | 644 (12.8) | 30 (8.7) | 188 (26.3) |
|  | 2016 | 222 (3.6) | 221 (4.4) | 0 (0) | 0 (0) |
|  | 2017 | 232 (3.8) | 232 (4.6) | 0 (0) | 0 (0) |
| Season | Hot, rainy (Dec–Mar) | 1637 (26.8) | 1320 (26.2) | 91 (26.4) | 226 (31.5) |
|  | Cool, dry (Apr–Aug) | 2909 (47.6) | 2399 (47.8) | 174 (50.4) | 336 (46.9) |
|  | Hot, dry (Sep–Nov) | 1561 (25.6) | 1326 (26.3) | 80 (23.2) | 155 (21.6) |

HIV, human immunodeficiency virus; ILI, influenza-like illness; SARI, severe acute respiratory infection

*The specimens were collected from 5,041participants with SARI, 205 participants, and 711 asymptomatic participants,

** No data were collected on paediatric ILI.

## **Supplementary Table 6.** Characteristics of participants with SARI, ILI, and asymptomatic control participants included in pathogen-attributable fraction analysis

|  | Paediatric | |  | Adult | | | |
| --- | --- | --- | --- | --- | --- | --- | --- |
|  | All  N=955 (%) | Asymptomatic  N=241 (%) | SARI  N=714 (%) | All  N=1915 (%) | Asymptomatic  N=476 (%) | ILI  N=345 (%) | SARI  N=1094 (%) |
| Sex |  |  |  |  |  |  |  |
| Male | 528 (55.3) | 112 (46.5) | 399 (55.9) | 941 (49.1) | 181 (38.0) | 133 (38.6) | 627 (57.3) |
| Female | 427 (44.7) | 129 (53.5) | 315 (44.1) | 974 (50.9) | 295 (62.0) | 212 (61.4) | 467 (42.7) |
| HIV status |  |  |  |  |  |  |  |
| Negative | - | - | - | 674 (35.2) | 213 (44.7) | 118 (34.2) | 343 (31.4) |
| Positive | - | - | **-** | 1237 (64.6) | 263 (55.3) | 227 (65.8) | 747 (68.3) |
| Missing | - | - | - | 4 (0.2) | 0 | 0 | 0 |
| Season  Hot, rainy (Dec-Mar) | 244 (25.5) | 59 (24.5) | 185 (25.9) | 453 (23.7) | 167 (35.1) | 91 (26.4) | 195 (17.8) |
| Cool, dry (Apr-Aug) | 540 (56.6) | 123 (51.0) | 417 (58.4) | 937 (48.9) | 213 (44.7) | 174 (50.4) | 550 (50.3) |
| Hot, dry (Sep-Nov) | 171 (17.9) | 59 (24.5) | 112 (15.7) | 525 (27.4) | 96 (20.2) | 30 (8.7) | 349 (31.9) |

ILI, influenza-like illness; SARI, severe acute respiratory illness.

*Seasonality designated using temperature and rainfall data from the Department of Climate Change and Meteorological Services, Malawi (www.metmalawi.com)
